# Supplementary material for: Psychosocial Interventions for Attention Deficit/Hyperactivity Disorder: A Systematic Review and Meta-Analysis by the CADDRA Guidelines Work GROUP
Source: Brain Sci. 2022 Aug 1;12(8):1023. doi: 10.3390/brainsci12081023 (PMC9406006; doi:10.3390/brainsci12081023)
Supplement: Supplementary file 1 [file brainsci-12-01023-s001.zip › brainsci-1797645-supplementary.pdf]

Supplementary Materials

Table S1. Inclusion and Exclusion Criteria Based on PICO.

Population	Include: Individuals (across the lifespan) with a diagnosis of attention deficit hyperactivity disorder<br>Exclude: None
Intervention	Include:<br>Psychoeducation<br>Therapeutic alliance / Health professional (Therapist)l factors<br>Motivational Interviewing<br>Cognitive Behavioral Therapy (CBT, Mindfulness-based CBT)<br>Psychotherapy (e.g., Dialectical Behavior Therapy, Trauma-focused, Emotion-focused, Eye movement desensitization and re-processing (EMDR), Play-based)<br>Social Skills Training<br>Behavior Therapy (e.g., Behavior Management/Modification, Reinforcement Schedules)<br>Parent/Caregiver training<br>Family interventions<br>School-based interventions/accommodations<br>Workplace interventions/accommodations<br>Mind-body interventions (e.g., Yoga, Mindfulness-based interventions, Meditation, Relaxation)<br>Healthy lifestyle management (sleep, nutrition, exercise)<br>Coaching (e.g., daily activity scheduling and organization)<br>EF Training/Remediation (8)<br>E-therapies<br>Exclude:<br>Peer-support/Tutoring/Mentoring<br>Cognitive training<br>Biofeedback<br>Nutritional supplements<br>Diet restrictions<br>Deep brain stimulation
Comparison	Include: No intervention, standard care (e.g., CBT), placebo<br>Exclude: Non-standard care
Outcomes	Outcomes to be assessed over the short (< 1 year) or long-term (> 1 year)<br>Outcome to be assessed by age (children under 11), adolescents (12-21), adults 21+<br><br>Include:<br>Primary<br>ADHD symptoms<br>Serious adverse events<br>Secondary<br>Quality of life<br>Functional status (a person's ability to do everyday tasks and activities)<br>Associated mental health problems (comorbidities)<br>Peer relationships<br>Family relationships<br>Academic outcomes, including school learning and progress<br>Care needs<br>Self-esteem<br>Mood/Emotion Regulation<br>Perceived control of symptoms<br>Risky behaviour (e.g., STIs, substance abuse, suicidality/self-harm, public health risk)<br>Oppositionality<br>Conduct problems<br>Parental confidence / Parenting skills<br>Treatment compliance/adherence<br>Motivation<br>Exclude:<br>Measurements not previously validated
Study Design	Include:

	Meta-analyses, systematic reviews, parallel RCTs (blinded and open), RCTs (any kind), controlled studies<br>Exclude:<br>Case-control, case-series, qualitative, letter to the editor, conference proceedings, cross-sectional

Advance Search Syntax:

Advanced search 1: "Attention Deficit Disorder with Hyperactivity"[Mesh] OR "Attention deficit"[TIAB] OR Hyperactivity [TIAB] OR Hyperactive[TIAB] OR ADHD[TIAB].

Advanced search 2: "Social Support"[Mesh] OR "Psychotherapy"[Mesh] OR "Motivational Interviewing"[Mesh] OR "Social Skills"[Mesh] OR "Healthy Lifestyle"[Mesh:NoExp] OR "Mentoring"[Mesh] OR "Distance Counseling"[Mesh] OR "Sleep"[Mesh] OR "Nutrition Therapy"[Mesh] OR "Exercise"[Mesh] OR Psychosocial[TIAB] OR "Social support"[TIAB] OR Psychotherapy[TIAB] OR psychotherapies[TIAB] OR Therapy[TIAB] OR therapies[TIAB] OR Psychoeducation[TIAB] OR Psychoeducational[TIAB] OR "Psycho-education"[TIAB] OR "Psycho-educational"[TIAB] OR Intervention[TIAB] OR interventions[TIAB] OR "Therapeutic alliance"[TIAB] OR "Motivational interviewing"[TIAB] OR Mindfulness[TIAB] OR "Eye movement desensitization and reprocessing"[TIAB] OR EMDR[TIAB] OR Training[TIAB] OR "Social skill"[TIAB] OR "social skills"[TIAB] OR "Behavior management"[TIAB] OR "Behaviour management"[TIAB] OR "Behavioral management"[TIAB] OR "Behavioural management"[TIAB] OR "Behavior modification"[TIAB] OR "Behaviour modification"[TIAB] OR "Behavioral modification"[TIAB] OR "Behavioural modification"[TIAB] OR "Reinforcement schedule"[TIAB] OR "reinforcement schedules"[TIAB] OR "School-based"[TIAB] OR Workplace[TIAB] OR Accommodation[TIAB] OR accommodations[TIAB] OR Lifestyle[TIAB] OR Sleep[TIAB] OR Nutrition[TIAB] OR nutritional[TIAB] OR exercise[TIAB] Or exercises[TIAB] OR "physical activity"[TIAB] OR "physical activities"[TIAB] OR "active living"[TIAB] OR coaching[TIAB] OR mentoring[TIAB] OR remediation[TIAB].

Advanced search 3"Meta-Analysis" [Publication Type] OR "Systematic Review" [Publication Type] OR "Randomized Controlled Trial" [Publication Type] OR "Controlled Before-After Studies"[Mesh] OR "Meta-analysis"[TIAB] OR "meta analysis"[TIAB] OR "Systematic review"[TIAB] OR "systematic reviews"[TIAB] OR "controlled trial"[TIAB] OR "controlled trials"[TIAB] OR "Controlled study"[TIAB] OR "controlled studies"[TIAB] OR RCT[TIAB].

Table S2. Detailed Data of Cognitive Behavioral Therapy Studies.

Author/ Year	Population	Design	Intervention	Comparator	N-randomized (m/f)	Principal outcome	Other outcomes	Instruments	Findings	Grade
Corbisiero et al 2018 [1]	Adults (18-49)	RCT	Individual CBT	Standard clinical management	43	ADHD symptoms	Stability of change at follow-up, impairment in daily life, quality of the psychotherapy	CAARS<br>CAARS-S:S<br>CAARS-O:L<br>WRAADDS<br>ADHD-SR<br>SDS<br>AAQol<br>SWE<br>RSES	Individual CBT does not outperform standard clinical management when added to medication.<br>Factor analyses were performed (principle axis method).<br>Factor "time": $F_{(1, 96)} = 83.49$, $p < 0.001$, $\eta2 = 0.723$<br>Factor "group": $F_{(1, 32)} = 0.01$, n.s.<br>Interaction effect of "group and time": F(1, 96) = 0.67, n.s.<br>→ Same pattern for ADHD subdomains: inattention, hyperactivity, impulsivity, emotional symptoms and for the two latent variables of impairment.<br><br>For the different ADHD scales: solution with one component found $\lambda = 3.21$), explaining 64.20% of the total variance.<br>→ Same procedure for each subdomain; solutions with one component were repeatedly found ($\lambda = 1.85–3.51$), explaining 56.36% of the variance in the subscales of inattention, 70.19% of hyperactivity, 61.71% of impulsivity, and 61.59% of emotional symptoms.<br><br>→ For impairment scales, solution with two factors was found ($\lambda_{i1} = 3.98$ and $\lambda_{i2} = 1.65$), explaining 56.25% of the variance. → On the first rotated factor all subscales of AAQoL (0.566–0.681) and the subscales of SDS for family (0.542) and work (0.398) loaded highly; subscale of SDS for leisure (0.318) and social contacts (0.319) loaded on both factors medium high. → Subscales of RSES and SWE loaded highly on second factor (0.641–0.862). Those two factors correlated slightly ($r = 0.362$).<br><br>Spearman's correlations for relationship between CBT process characteristics and changes in symptom and impairment levels.<br>→ Emotion in therapy: high correlations for inattention ($\varrho = −0.57$, $p < 0.01$), hyperactivity ($\varrho = −0.60$, $p < 0.01$), and emotional symptoms ($\varrho = −0.60$, $p < 0.01$), but not for impulsivity ($\varrho = −0.28$, n.s.).	
Dittner et al 2017 [2]	Adults (18-65)	RCT	Individual CBT	Treatment as usual	60	1)ADHD symptoms<br>2)Functional impairment	Depression sx, anxiety, global distress, patient satisfaction.	CSS<br>WSAS<br>HADS<br>CORE-OM<br>CGI (improvement and satisfaction)	Individual CBT showed a large improvement in ADHD symptoms and functioning when compared to treatment as usual.<br>CSS: ES =−1.31 (P < 0.001)<br>WSAS: ES = -0.82 (P = 0.003)<br><br>CGI: Odds ratio (OR) for participant-rated CGI improvement for CBT vs TAU = 23.1 (P < 0.001).<br>CGI Satisfaction: OR = 23.2 (P < 0.001).<br>→ For blind assessors, OR was not computable as 0 participants were rated as improved in the TAU group.<br><br>CSS Informant: Moderate but non-significant benefit of CBT at 42 weeks (standardised ES = −0.38) and 30 weeks (ES = −0.42).<br><br>HADS: HADS Anxiety scores lower at 42 weeks (ES = −0.6, P = 0.012) and at 30 weeks (ES = −0.62, P = 0.015) for CBT vs. TAU.<br>HADS Depression scores lower at 42 weeks (ES = −0.66, P = 0.002) and 30 weeks (ES = −0.61, P = 0006).<br><br>CORE: For CORE subdomains, improvement for all domains in CBT group compared to TAU at 42 and 30 weeks.<br>→ Only Problems and Wellbeing showed a statistically significant decrease.<br>→ Moderate decrease for Problems (42 weeks, ES = −0.59, P = 0.025 and 30 weeks, ES = −0.58, P = 0.023)<br>→ Large decrease for Wellbeing (42 weeks ES = −1.03, P = 0.02; 30 weeks ES = −1.01, P = 0.02).<br>→ CORE Functioning and Risk, effects small, below 0.3 at both 30 and 42 weeks.	
Safren et al 2010 [3]	Adults (18-65)	RCT	Individual CBT	Relaxation with educational support (attention-matched control)	86	1)ADHD symptoms<br>2)Maintenance of change (if any) in ADHD symptoms	N/A	CGI (rater report)<br>ADHD-rating scale (rater report)<br>CSS (self-report)	Individual CBT improved ADHD symptoms; these gains were maintained over time.<br>ADHD-rating scale:<br>Estimated parameter for treatment effect = −4.63 [95% CI, −8.30 to −0.96]; $t_{23.73}$=−2.36, P=.02; d=0.60.<br>Proportion of responders in CBT group compared to control group = 67% vs 33%; OR, 4.29 [95% CI, 1.74 to 10.58]; P=.002	

									→ Slope of score over time (posttreatment, 6-month follow-up and 12-month follow-up assessments) of those who were assigned to the CBT condition and responded or had a partial response did not significantly differ from zero, indicating maintenance of gains: β = −0.12; 95% CI, −0.41 to 0.18; P = .41.<br>→ Slope for blinded assessor-rated scale did not change statistically over time (posttreatment, 6-month, 12-month assessment; β=−0.17 [95% CI, −0.47 to 0.13], P=.27) and did not differ by condition (β = 0.08 [95% CI, −0.33 to 0.49], P = .69).<br><br>CGI:<br>Treatment effect −0.53 [95% CI, −1.01 to −0.05]; $t_{24.31}$=−2.29, P=.03; d=0.53<br>Proportion of responders compared to control group = 53% vs 23%; OR, 3.80 [95% CI, 1.50 to 9.59]; P=.01<br>→ Slope of score over time (posttreatment, 6-month follow-up and 12-month follow-up assessments) of those who were assigned to the CBT condition and responded or had a partial response did not significantly differ from zero, indicating maintenance of gains: β = 0.01 [95% CI, −0.03 to 0.05]; P = .59<br>→ Slope for blinded assessor-rated scale did not change statistically over time (posttreatment, 6-month, 12-month assessment; β = 0.01 [95% CI, −0.03 to 0.05], P = .73) and did not differ by condition (β = 0 [95% CI, −0.05 to 0.06], P = .97).<br><br>CSS (self-report):<br>Scores: β=−8.18 [95% CI, −12.41 to −3.96]; P<.001; however, this was qualified by interaction of treatment condition by time (β = −0.15 [95% CI, 0.04 to 0.27]; P = .01).<br>→ Slope of score over time (posttreatment, 6-month follow-up and 12-month follow-up assessments) of those who were assigned to the CBT group and responded or had a partial response did not significantly differ from zero, indicating maintenance of gains: β = 0.05 [95% CI, −0.04 to 0.15]; P = .26<br>→ Slopes for each treatment condition separately indicated increasing slope for CBT group: β=0.08 [95% CI, 0 to 0.15]; P=.04 (but small magnitude of these effect reveals change of limited clinical significance).	
Gu et al 2016 [4]	Adults (19-24)	RCT	Individual MBCT	Wait list	54	1)ADHD symptoms<br>2)Anxiety and depression	Academic performance, mindfulness levels, neuropsychological performance, treatment gains for MBCT at 3 month follow-up assessment	CAARS-S (3 subdomains: hyperactivity/impulsivity, inattention, ADHD index)<br>BDI-2<br>GPA<br>MAAS<br>ANT<br><br><br>RM ANOVA	In intent-to-treat analyses, participants who received MBCT showed an overall trend toward lower inattentive symptoms.<br>→ F(2, 52) = 9.380, p = .003, partial η2 = .153.<br><br>CAARS (ADHD symptoms):<br>MBCT showed significantly greater improvement than wait list on hyperactivity/impulsivity symptoms and ADHD index.<br>→ Among those responding to treatment, 16 (57%) showed positive response after MBCT, whereas six (23%) showed positive response after WL, χ2(1) = 3.24, p = .07.<br>At follow-up, 20 (71%) MBCT participants showed recovery, compared with 8 (31%) of wait list participants, χ2(1) = 4.46, p = .04.<br><br>Anxiety/depressive symptoms (BDI):<br>Repeated-measures (RM) ANOVA for BDI (anxiety): significant time effects, F(2, 52) = 5.890, p = .019, partial η2 = .102.<br>But no significant time effects on depressive symptoms: F(2, 52) = 2.437, p = .125, partial η2 = .045.<br>MBCT group: significant change in depressive symptoms at post-treatment and follow-up.<br>Academic performance (GPA):<br>No significant change in GPA when compared with wait list group, F(2, 52) = 0.366, p = .548, partial η2 = .007.<br><br>MAAS (mindfulness):<br>RM ANOVA for mindfulness: significant improvement at overall post-treatment and follow-up.<br>→ F(2, 52) = 9.965, p = .003, partial η2 = .161<br>→ Based on planned contrasts, MBCT outperformed wait list both at post-treatment, F(1, 52) = 11.831, p = .001, d = 1.06, and at follow-up, F(1, 52) = 10.862, p = .001, d = 1.30.<br><br>ANT (Neuropsychological performance):<br>MBCT group showed trend toward greater improvement on normalized reaction time (RT) and error score (ES) network data in the alerting network, versus those in wait list group.<br>MBCT also outperformed wait list both on normalized RT and ES network data in the orienting network.	0.89

									→ But MBCT did not significantly outperform wait list in the conflicting network on normalized RT network data: $F(2, 52) = 0.069$, $p = .793$, partial $\eta 2 = .001$, or ES network data, $F(2, 52) = 1.480$, $p = .229$, partial $\eta 2 = .028$.	
Emilsson et al 2011 [5]	Adults	RCT	Group and individual CBT-R&R2ADHD	Treatment as usual	54	ADHD symptoms	Anxiety, depression, emotional control, social functioning and antisocial behaviour	K-SADS-PL, ADHD section (rater report)<br>CGI (rater report)<br>CSS (self-report)<br>BAI (self-report)<br>BDI (self-report)<br>RATE-S (self-report) with 4 subscales (ADHD symptoms, emotional control, anti-social behaviour, social functioning.	Findings support effectiveness of R&R2ADHD in reducing ADHD symptoms and comorbid problems.<br><br>K-SADS ADHD (rater report):<br>→ At end of treatment: $F(1,31) = 11.02$, $p < .01$; Cohen's d: 1.03<br>→ At 3 month follow-up: $F(1,18) = 7.60$, $p < .05$; Cohen's d: 1.17<br><br>CGI (rater report):<br>→ At end of treatment: No significant difference between the two groups ($p = .06$)<br>→ At 3 month follow-up: $F(1,18) = 9.16$, $p < .05$; Cohen's d: 1.31<br><br>CSS (self-report):<br>a) Total:<br>→ At end of treatment: $F(1,32) = 10.45$, $p < .01$; Cohen's d: 0.76<br>→ At 3 month follow-up: $F(1,29) = 17.36$, $p < .001$; Cohen's d: 1.08<br>b) Inattention:<br>→ At end of treatment: $F(1,32) = 8.73$, $p < .05$; Cohen's d: 0.94<br>→ At 3 month follow-up: $F(1,29) = 10.70$, $p < .01$; Cohen's d: 1.15<br>c) Hyperactivity/impulsivity:<br>→ At end of treatment: $F(1,32) = 7.27$, $p < .05$; Cohen's d: 0.32<br>→ At 3 month follow-up: $F(1,29) = 20.30$, $p < .001$; Cohen's d: 0.58<br><br>BAI (self-report):<br>→ At end of treatment: no significant difference between the two groups ($p = .46$).<br>→ At 3 month follow-up: $F(1,29) = 4{,}61$, $p < .05$; Cohen's d: 0.83<br><br>BDI (self-report):<br>→ At end of treatment: no significant difference between the two groups ($p = .052$)<br>→ At 3 month follow-up: $F(1,29) = 5.86$, $p < .05$); Cohen's d: 1.32<br><br>RATE-S (self-report):<br>a) Total score:<br>→ At end of treatment: no significant difference was found between the two groups.<br>→ At 3 month follow-up: $F(1,28) = 14.77$, $p < .001$; Cohen's d: 1.46<br>b) ADHD scale:<br>→ At end of treatment: no significant difference between the two groups ($p = .16$)<br>→ At 3 month follow-up: $F(1,28) = 11.83$, $p < .01$; Cohen's d: 1.08<br>c) Emotional control scale:<br>→ At end of treatment: No significant difference was found between the two groups ($p = .48$)<br>→ At 3 month follow-up: $F(1,28) = 6.35$, $p < .05$); Cohen's d: 1.12<br>d) Social functioning scale:<br>→ At end of treatment: no significant difference was found between the two groups<br>→ At 3 month follow-up: $F(1,28) = 10.88$, $p < .01$; Cohen's d: 1.24<br>e) Antisocial scale:<br>→ At end of treatment: $F(1,31) = 4.75$, $p < .05$; Cohen's d: 0.84<br>→ At 3 month follow-up: $F(1,29) = 7.28$, $p < .05$; Cohen's d: 0.89	
Schonberg et al 2014 [6]	Adults (19-53)	RCT	MBCT (Group)	Wait list	50<br>44 "statistically viable" (23 F, 21 M)	1) Error processing<br>2) Conflict monitoring<br>3) Inhibitory control	Inattention and hyperactivity-impulsivity ADHD symptoms, psychological distress, social functioning, mindfulness skills	CAARS-S:SV (self-rating)<br>Outcome questionnaire (OQ)<br>KIMS (Kentucky Inventory of Mindfulness)<br>EEG data (ERPs-event related potentials)-segmented into a) response-locked false alarms to NoGo stimuli (FA), b) response-locked correct hits to Go stimuli (CH), c) stimulus-locked NoGo trials (NoGo-T), d)	Behavioural data:<br>A main effect of Condition was evident for task accuracy scores ($F(3, 120) = 92.828$, $p < .0001$), and also for RTs ($F(1, 40) = 5.724$, $p = .022$). Despite no significant Group effect/Time x Group interaction, number of FAs significantly decreased pre-to-post in the MBCT group alongside a significant slowing in RTs, not present in the wait list (WL).<br><br>ERN/CRN:<br>Main effects of Condition ($F(1, 41), = 19.059$, $p < .0001$) and Site ($F(2, 82) = 5.555$, $p = .01$) were evident, reflecting higher ERN amplitudes compared to CRN. There was no main effect of Group ($p = .85$), although a Time x Condition x Site z Group ($F(2, 82) = 3.357$, $p = .05$) interaction indicated overall ERN amplitude attenuation pre-to-post MBCT, contrary to amplitude increase at Fz and Cz in the WL. However, follow-up post hoc t-tests revealed such amplitude changes were not significant.	

								stimulus-locked Go trials (Go-T).<br>Response-locked evoked potentials (ERN), correct-related negativity (CRN)	Despite no main effect of Medication status (p = .18), a trend Condition x Site x Medication (F(2, 82) = 3.035, p = .07) interaction was found. Post-hoc tests showed overall medicated patients had higher ERN amplitudes compared to non-medicated, significantly so at Cz only (F(1, 42) = 7.370, p = .01). A Group x Medication post hoc data-split indicated there were no significant pre-to-post differences in either medicated or non-medicated patients for either group, aside for medicated patients exposed to MBCT showed a significant decrease in ERN amplitude at Cz (t(13) = -2.323, p = .04) [-9.84(8.4) μV to -7.27(6.7) μV).<br>Taking latency, main effects/interaction of Site (F(2, 82) = 6.490, p = .002), Group (F(1, 41) = 5.452, p = .03), and Condition x Site (F(2, 82) = 3.145, p = .05) were found. Although no main effect of Medication (p = .85), a Site x Medication (F(2, 82) = 3.695, p = .03) interaction revealed faster ERN/CRN latencies from pre-to-post in both MBCT and WL groups, regardless of medication status.<br>Post-hoc Group x Medication tests were not significant, except for medicated patients undergoing MBCT showed significantly reduced ERN latency at Cz (t(13) = 3.821, p = .002) [71.6(19.6)–59.9(23.9) ms].<br><br>Pe/Pc:<br>Main effects of Time (F(1, 41) = 5.573, p = .02), Condition (F(1, 41) = 36.276, p < .0001), and Condition x Site (F(2, 82) = 3.552, p = .033) indicated amplitudes increased pre-to-post in both groups, and significantly so at FCz for Pe (t(23) = -2.613, p = .02) [9.75(5.7)–13.99(7.3) lV] in the MBCT group (Fig. 1 and Fig. 2), contrary to Fz (t(19) = -2.809, p = .01) [8.5(3.5)–12.4(5.3) μlV], and Cz (t(19) = -2.139, p = .05) [10.1(3.7)–12.09(5.1) μV] in the WL. There were no significant findings for Pe/Pc latency measures.<br><br>NoGo-N2:<br>Time (F(1, 41) = 14.241, p = .001), Site (F(1, 41) = 8.071, p < .0001) main effects, and Site x Group (F(3, 123) = 3.011, p = .033) interaction were evident. As there was a trend Time x Condition x Group (F(1, 41) = 3.204, p = .081) interaction, posthoc tests were conducted, indicating general increase in Go and NoGo-N2 amplitudes across sites in the WL, significantly so for Go-N2 at Fz (t(19) = 3.902, p = .001) [-2.79(2.6) to -4.20(2.9) μV], and Pz (t(19) = 2.164, p = .04) [.436(2.3) to -.115(2.0) μV]. Conversely, amplitude attenuation was evident for NoGo-N2 pre-to-post MBCT at Fz (p = .86) [-2.22(3.6) to -2.11(3.1) μV], Cz, (p = .47) [-2.30(4.3) to -2.02(3.8) μ V], and Pz (p = .51) [<br>1.64(2.8) to<br>1.34(2.9) lV], compared to overall increase for Go-N2, significantly so at FCz (t(23) = 2.095, p = .05) [<br>1.43(2.8) to<br>2.18(2.7) lV]. No main effects of Group (p = .64), or Medication (p = .29).<br>Examining N2 latency, Site (F(3,57) = 3.406, p = .05) and Time -<br>Site<br>Group (F(3,57) = 3.50, p = .04) effects showed decreased latency (faster peaking) for NoGo-N2 pre-to-post MBCT, compared to an overall slowing in WL, and slowing of Go-T for both groups.<br>Although, latency change in both groups were marginal, not significant.<br><br>NoGo-P3:<br>Higher amplitudes were yielded for NoGo-P3 compared to Go-P3 in both groups (Condition: F(1, 41) = 60.080, p < .0001). Site (F(3, 123) = 3.289, p = .02), Condition x Site (F(3,123) = 11.631, p < .0001), Condition x Site x Group (F(3, 123) = 2.696, p = .05), and Time x Site x Group (F(3, 123) = 2.514, p = .06) and Time x Condition x Group (F(1, 41) = 3.220, p = .08) trends, showed significant increase in Go-P3 (t(23) = -2.986, p = .007) [5.04(2.7) to 5.96(2.8) μV], and NoGo-P3 (t(23) = -2.502, p = .02) [8.82(4.4) to 10.10(4.1) μV] amplitudes at Pz pre-to-post MBCT, contrary to parietal (Pz) decrease in the WL for Go-P3 (p = .42) [6.14(2.2) to 5.76(2.5) μV], and NoGo-P3 (p = .40) [9.69(2.8) to 9.13(3.7) μV]. Taking latency, Time (F(1, 41) = 4.048, p = .05), Condition (F(1,41) = 4.594, p = .04), Site (F(3, 123) = 7.673, p < .0001), and Condition x Site (F(3, 123) = 12.073, p < .0001) interaction show overall increase in Go/NoGo-P3 latency in both groups.	

									Clinical effects:<br>Examining the CAARS-SV, main effects of Group (F(1, 38) = 4.713, p = .04), Domain (F(2, 76) = 28.884, p < .0001), further to Time - x Group (F(1, 38) = 9.248, p = .004), and Time x Domain x Group (F(2, 76) = 5.227, p = .01) interactions, showed reduced 'inattention' (t(22) = 4.891, p < .0001), 'hyperactivity/impulsivity' (t(22) = 3.161, p < .0001), and global ADHD index (t(22) = 4.239, p < .0001) symptoms pre-to-post MBCT exclusively.<br>Examining the outcome questionnaire (OQ-45.2), main effect of Domain (F(2, 76) = 28.885, p < .0001), and Time x Group (F(1, 38) = 4.924, p = .033) interaction indicated amelioration in 'symptom distress' (t(22) = 2.392, p = .03), 'social role' (t(22) 2.265, p = .03), and global score (t(22) = 2.964, p = .007), in the MBCT group only.<br><br>Mindfulness skills:<br>Main effect of Domain (F(3, 114) = 3.338, p = .03), and Time x Group (F(1, 38) = 22.845, p < .0001) interaction, reflected improved mindfulness skills for all domains in the MBCT group pre-to-post; 'observe' (t(22) = -3.301, p = .003), 'describe' (t(22) = -2.459, p = .022), 'act-with-awareness' (t(22) = -4.350, p < .0001), and 'act-without-judgement' (t(22) = -2.681, p = .01). As expected, no significant changes were evident in the WL group.<br><br>Correlational analyses:<br>Increases in act-with-awareness on the KIMS correlated with decreases in CAARS global scores (r(23) = -.832, p < .001), 'inattention' (r(23) = — .618, p = .002), and 'hyperactivity/impulsivity' (r(23) = — .893, p < .001) subdomains in the MBCT group. Likewise, increases in KIMS act-without-judgement correlated with decreases in global CAARS (r(23) = — .632, p = .001), 'inattention' (r(23) = — .632, p = .001), and 'hyperactivity/impulsivity' (r(23) = — .533, p = .009) exclusive to MBCT. Conversely, CAARS 'inattention' and KIMS 'observe' were positively correlated in the WL (r(19) = .537, p = .02).<br>Examining mindfulness/CAARS and ERP measures; no significant correlations pertained to the ERN in either group, nor for the Pe in the WL. However, reduction in CAARS 'hyperactivity/impulsivity' correlated to increased Pe amplitudes at Fz (r(23) = -.456, p = .03) and Cz (r(23) = -.453, p = .03) pre-to-post MBCT only, further to increased KIMS act-with-awareness associated with increased Pe amplitude at Fz (r(23) = .491, p = .02).<br>Increased P3 amplitudes correlated to increased mindfulness skills pre-to-post MBCT only. KIMS 'describe' with the Go-P3 at Cz (r(23) = .483, p = .02), and FCz (r(23) = .416, p = .05). act-without-judgement and the Go-P3 at Cz (r(23) = .513, p = .01), and Pz (r(23) = .545, p = .007), further to the NoGo-P3 at Cz (r(23) = .466, p = .03), and Pz (r(23) = .484, p = .02). Furthermore, reduced scores on the CAARS-inattention subdomain and increased amplitudes at Cz for Go-P3 (r(23) = -.429, p = .046), and NoGo-P3 (r(23) = -.476, p = .02), were evident.	
Solanto et al. 2010 [7]	Adults (18-65)	RCT	Group CBT	Support group	88 (30 M/58F)	ADHD symptoms	Anxiety, depression, self-esteem	AISRS-IN (Adult ADHD Investigator Symptom Rating Scale, inattention symptoms) and TMOP (subscale of the AISRS-IN, five items reflecting time management, organization and planning)<br>CAARS-Self-Inattention Scale<br>HARS (Hamilton Anxiety Rating scale)<br>Brown ADD scale<br>BRIEF-A (Behavior Rating Inventory of Executive Function–Adult Version)<br>BDI-II<br>RSEI (Rosenberg Self-Esteem Inventory)	The pattern of treatment contrasts indicated that the larger (more severe) the score at baseline, the greater the differential improvement with MCT; this occurred whether the data were analyzed with or without non-completers and medication changers (interaction coefficients 0.66 and 0.72, respectively).<br><br>AISRS and CAARS:<br>MCT group improved by 5.0 points, whereas Support improved by 2.3 points, a difference between groups of 2.7 (95% CI = 0.9, 4.6; p<0.005) or 56% of the overall standard deviation of the change score (4.8).<br>→ Same pattern (i.e., greater change in MCT vs. Support), was evident on the AISRS-TMOP and the CAARS-Observer-Inattention.<br>Responders:<br>→ AISRS-IN: 19 participants (42.2%) in the MCT group were responders, compared to only 5 (12%) in Support ($\chi2$ = 10.38, df=1, p=.002).<br>→ Conners-Self-Inattention: 24 (53%) in the MCT group and 12 (28%) in the Support group were responders ($\chi2$ = 5.88, df=1.p=.018).<br>*Logistic regression results revealed a significant effect of Treatment Group on responder status (odds ratio of 5.41; 95% CI = 1.77,16.55) favoring MCT.<br><br>Brown and ON-TOP:<br>Significant change from pre- to post-treatment for Support and MCT.	

								ON-TOP (On Time Management Organization and Planning)<br>Conners-Observer (Conners-Observer Report: Long Version)<br><br>General Linear Modeling	→ However, the change score difference between groups was not significant (Brown) or only marginally significant (ONTOP).<br><br>BRIEF-A Metacognitive Index:<br>Marginally significant greater improvement in MCT compared to Support.<br>→ Change in MCT but not Support was significant.<br><br>BDI-II, RSEI, and HARS:<br>No differences observed between treatment groups (MCT and Support) in pre- to post-change scores for depression (Beck), self-esteem (Rosenberg) or anxiety (Hamilton).<br>→ Confidence intervals for change scores for each treatment group separately showed no significant effects for any of these outcome variables.<br>→ Results of ANOVA showed that for participants with a concurrent axis I mood disorder mean Beck scores decreased from 17 to 13, yielding a significant main effect of Time (pre- to post-treatment), F (1,24) =4.998, p=.035, but no interaction with treatment condition.<br>→ The same analysis was done for those with a current anxiety disorder vis-à-vis scores on the Hamilton with no significant results.<br><br>Adjustments:<br>Mixed model analyses of variance were run to adjust for intra-cluster correlation using Group, Therapist, and Cohort as clusters. → Therapist consistently did not account for any intra-cluster correlation.<br>→ Adjusting for Group and Cohort simultaneously as random variables did not affect the significance of the noted treatment effects.	
Solanto et al. 2018 [8]	Adults (18-65)<br>Same population as above	RCT (data reanalysis from 2010 study; see above)	Group CBT (older vs younger adults)	Support group (older vs younger adults)	88 (30 M/58F), 26 older (over 50 yrs old) and 55 younger (less than 50 yrs old)	Inattentive symptoms	Attention<br>Executive dysfunction<br>Comorbidity	AISRS-IN (ADHD Investigator Symptom Rating Scale, Inattention items)<br>CAARS-S Inatten/Mem scale<br>Brown ADD scales<br>BRIEF-A (Behavior Rating Inventory of Executive Function-Adult Version)<br>ON-TOP (On Time Management Organization and Planning questionnaire<br>BDI-II (Beck Depression Inventory-II)<br>Rosenberg Self-Esteem Inventory<br><br>SIG-H (Structured Interview Guide for the Hamilton Anxiety Rating Scale, independent evaluator)<br>CAARS-Observer Report: Long Version (CAARS-Obs) completed by spouse/partner/family member/friend	s	

Table S3. Detailed Data of Physical and Mind Body Studies.

Author/ Year	Population	Design	Intervention	Comparator	N (m/f)	Principal outcome	Other outcomes	Instrum-ents	Findings					Grade
									Inattention	Hyperactivity	Impulsivity	Overall	Other	

Cerrillo-Urbina et al 2015 [9]	children &/or adolescents (6–18)	MA RCT	Weekly Yoga, Short-term aerobic exercises (6–10 weeks), based on several aerobic intervention formats	no PE	249	inattention, hyperactivity impulsivity	anxiety, executive function, social disorders, cognitive performance	(Conners or DSM in any of its editions); and (vi) language (all accepted)	Moderate - large effect	moderate -large effect	Moderate- large effect		Moderate- large effect on anxiety, executive function, social disorders Less evidence for yoga	Low Small number of studies heterogeneity of outcome measures
Zang 2019 [10]	Children &/or adolescents (8.29 – 16) One study: 21 RCT, observational cohorts	MA Included observantional and RT	Various physical exercises	Various	574	aggressive behaviors, perseverative errors, internalized/ externalized problems, social, thought problems		Various	NS improvement : (WMD: -0.22; 95% CI: [-0.51 – 0.08], P=.15)	NS improvement : Hyperactive/ impulsive symptoms (WMD: -0.01; 95% CI: [-0.32 – 0.29], P=.93)	NS improvement : Hyperactive/ impulsive symptoms (WMD: -0.01; 95% CI: [-0.32 – 0.29], P=.93),		Sig improved: Anxiety, depression (WMD: -1.84; 95% CI: [-2.65 – (-1.03)], P=.00001). thought problems (WMD: -3.49; 95% CI: [-5.51 – (-1.47)], P=.0007), social problems (WMD: -5.08; 95% CI: [-7.34 – (-2.82)], P=.0001), aggressive behaviors (WMD: -3.90; 95% CI: [-7.10 –(-0.70)], P=.02) Strength & agility (WMD: 5.69; 95% CI:[1.13 – 10.25], P=.01), Stroo color- word test (WMD: 6.67; 95% CI: [4.21 – 9.13], P=.00001)	Very low
Kang et al. 2011 [11]	adolescents diagnosed as ADHD, Department of Psychiatry of Chung Ang University Medical Center, 8.6, 8.4 yo Korean boys middle-class	RCT	90 min sessions of sports therapy 2x/ wk, 6 wk	The educational intervention 6 wk	28	attention problems, hyperactivity scores	executive functions, social skills	Korean DuPaul's ADHD Rating Scale (K-ARS-PT). Digit Symbol Test The Trail Making Test part B (TMT B). The Social Skills Rating System	greater improvements in K-ARS-PT-inattention (F(1, 26) = 10.41,p < 0.01)	No significant difference in change in K-ARS-PT-hyperactivity (F(1, 26) = 1.96, p = 0.17)		Greater improvements in the K-ARS-PT (F(1, 26) = 4.81, p = 0.04).	Executive Function: Scores in the sports group increased (z = 2.66, p < 0.01), unchanged in education group (z = 0.01, p = 0.99). Greater reduction in TMT-B performance time in sports compared to education group (F(1, 26) = 4.86, p = 0.04). Social Skills: Cooperativeness scores in the sports compared to education group. No difference in changes in assertiveness (F(1, 26) = 1.15, p = 0.29) or self-control scores (F(1,26) = 1.28, p = 0.27)	moderate
Meßler et al. 2016 [12]	8 to 13 years, ADHD (F90; F90.0; F90.1) 28 healthy boys with ADHD Five boys in HIIT, four in TRAD group were taking medication	RCT, 3 weeks	Child Physical HIIT Training, 4X 4 min, 3X/ wk, 3 wks	60-min sessions of ball and team games, court sports, and climbing at low-to-moderate intensity 1x/ wk	28	Motor skills	Quality of Life, Attention, hyperactivity	Movement Assessment Battery for Children II (M-ABC-II), questionnaire for external evaluation by the guardians (FBB-HKS) form for self-assessment by the children (SBB-HKS),DIS-YPS-II [German rating scale for symptom lists	Attention improved following HIIT from 'very noticeable' to "noticeable."	No difference between groups, or pre/post change	No difference between groups, or pre/post change	Parents : HIIT improved, TRAD reduced competence (p ≤ .01; ηp 2 = .37; F = 15.113)	Parent eval.: Emotional well-being reduced in both groups (p = .04; ηp2 = .16; F = 4.775), friends subscale higher after HIIT, lower after TRAD (p ≤ .01; ηp 2 = .23; F = 7.555) , QOL higher after HIIT (p ≤ .01; ηp 2 = .24; F = 7.975), lower after TRAD Boys: self-esteem better after HIIT(p ≤ .01; ηp 2 = .25; F = 8.792), worse after TRAD (p ≤ .01; ηp 2 = .30; F = 11.019).	low

								for hyperkinetic and conduct or oppositional defiant disorders based on ICD-10 criteria], KINDL questionnaire (health-related quality of life)					Parents, Boys: No changes in physical well-being.<br>same for both groups and time-points (p = .11; ηp2 = .10; F = 2.829).<br>No change in QOL in either group (p ≤ .12; ηp2 = .00; F = 2.576)<br>Motor Skills<br>HIIT, not TRAD improved manual dexterity (p ≤ .05; ηp2= .15; F = 4.458); ball skills (p = .03; ηp2 = .18; F = 5.686), total score (p = .01; ηp2 = .23;F = 7.688).<br>No differences in static and dynamic balance (p- .26; ηp2 = .05 F = 1.352).	

Table S4. Detailed Data of Caregiver Interventions Studies.

Author/year	Population	Design	Intervention	Comparator	n (m/f)	Principal outcome	Other outcomes	Instruments	Findings	Grade
Rimestad et al., 2019 [13]	Preschool children 2.5 to 6 years with ADHD	Meta-analysis	Parent training programs	No or minimal intervention groups (e.f., waitlist, treament as usual, minimal intervention, or attention control)	1003	Child ADHD symptoms	Child conduct problems , negative parenting	Various	Post-intervention<br>ADHD symptoms:<br>The ES of PT on parent-reported ADHD symptoms compared with WL, PLA, or TAU (n =15) was significant and moderate (0.51, CI = [0.33, 0.69], p < .001).<br>The ES of PT on independently assessed ADHD symptoms (n = 9) was<br>insignificant and small (0.12, CI = [−0.12, 0.36], p = .325).<br><br>Conduct problems:<br>The ES of PT on parent-reported conduct problems (n = 13) was small to moderate and significant (0.44, CI = [0.17-0.70], p = .001). Analysis of independently assessed child conduct problems (n = 5) revealed a small and insignificant ES (0.31, CI = [−0.07–0.69], p = .117)<br><br>Negative parenting.<br>The ES of PT on self-reported negative parental style (n = 8) was significant and moderate to large (0.63, CI = [0.32– 0.93], p < .001). The ES on independently assessed negative parental style (n = 9) was significant and small (0.33, CI = [0.13–0.53], p = .001)<br><br>Follow-up<br><br>ADHD symptoms.<br>The ES of PT on parent-rated ADHD symptoms from post-treatment to follow-up (n = 8) was slightly positive, and marginally significant (0.07, CI =[−0.01, 0.15], p = .059).<br><br>Conduct problems.<br>The ES of PT on parent-rated conduct problems from post-treatment to follow-up (n = 8) was slightly positive, but insignificant (0.07, CI = [0.01, 0.15], p = .103).<br><br>Negative parenting.<br>The ES of PT on parent-rated negative parenting from post-treatment to follow-up (n = 5) was small, but positive and marginally significant (0.12, CI =[−0.01, 0.24], p = .059).	
Mulqueen et al., 2015 [14]	Preschool children < 6 years with ADHD	Meta-analysis	Behavioral interventions:<br>Six trials :	Six trials compared a parent training program with a wait-	399	Child ADHD symptoms	N/A	Child Behavior Checklist, Behavior Assessment	Meta-analysis demonstrated a significant benefit of behavioral parental interventions compared with control conditions (SMD = 0.61, 95% confidence interval [CI] = [0.40, 0.83], z = 5.6, p < .001).	

			parent training vs wait-list/treatment as the usual.<br>One trial : combined parent and child training vs waitlist.<br>One trial : combined parent training and school consultation vs community treatment.	list/treatment as the usual condition, one trial compared a combined parent and child training program with a waitlist condition, and one trial compared a combined parent training and school consultation program with a community treatment control condition.				System for Children, Conners' Parent Rating Scale, Parental Account of Children's Symptoms, and Preschool and Kindergarten Behavior Scale.	Behavioral parental interventions also demonstrated a significant benefit compared with control conditions when a random-effects model was utilized as opposed to a fixed-effects model (SMD =0.65, 95% CI = [0.26, 1.05], z = 3.3, p = .001).	
Herbert et al., 2013 [15]	Preschool children with ADHD	RCT	BPT : The Parenting Your Hyperactive Preschooler program	Waitlist	31 (23/8)	Mother reports for:<br>1) Child ADHD, ODD symptoms, emotional dysregulation<br>2) Self-report of parenting, Parental emotion socialization	Father reports and Observational data for the main outcomes	BASC 2-PRS ;<br>Disruptive Behavior Rating Scale (DBRS) ;<br>Emotion Regulation Checklist (ERC);<br>The Parenting Scale ; The Coping With Children's Negative Emotion Scale (CCNES) ; Audiotaped Assessment of Parent-Child Interaction	Compared to WL mothers, PT mothers reported significantly less child inattention, hyperactivity, oppositional defiance, and emotional lability; were observed using significantly more positive and less negative parenting; and reported significantly less maternal verbosity and unsupportive emotion socialization practices.<br><br>Ratings of audiotaped observations :<br>did not reveal significant differences between groups in children's misbehavior or negative affect. Mothers who participated in the PT group were rated as engaging in more positive parenting and expressing less negative affect, controlling for observed childbehavior, compared to mothers in the WL group. These differences represented large-sized effects.<br><br>Father reports:<br>At posttest, fathers in the PT group rated their children significantly lower on the DBRS inattentive subscale and hyperactive/impulsive subscale, compared to pretest reports. The difference in DBRS hyperactivity/impulsivity represented a large-sized effect, and the differences in inattention represented a medium-sized effect. Similar to mothers, fathers reported a significant and large-sized decrease in unsupportive emotion socialization practices, but did not report a significant change in supportive emotion socialization practices.	
Sonuga-Barke et al., 2018 [16]	Preschool children between 2 years 9 months and 4 years 6 months old with ADHD	RCT	BPT: New Forest Parenting Program (NFPP) or Incredible Years (IY)	TAU	290 (208/82)	Parent ratings of child's ADHD symptoms	Teacher ratings (SNAP-IV) and direct observations of ADHD symptoms and parent/teacher ratings of conduct problems<br><br>Cost analysis	- Swanson Nolan and Pelham (SNAP)-IV–Parent and Teacher Scales ;<br>- Eyberg Child Behaviour Inventory (ECBI);<br>- Directly Observed Attention (DOA) derived using direct observation of 5-min episodes of child solo play on the 'Little People Animal Sounds Zoo';<br>- Client Service Receipt Inventory (CSRI);<br>- General Health Questionnaire (GHQ).	NFPP and IY did not differ on parent-rated SNAP-IV, ADHD combined symptoms [mean difference − 0.009 95% CI (− 0.191, 0.173), p = 0.921] or any other measure.<br><br>Small, non-significant, benefits of NFPP over TAU were seen for parent-rated SNAP-IV, ADHD combined symptoms [− 0.189 95% CI (− 0.380, 0.003), p = 0.053]. NFPP significantly reduced parent-rated conduct problems compared to TAU across scales (p values< 0.05).<br><br>No significant benefits of IY over TAU were seen for parent-rated SNAP, ADHD symptoms [− 0.16 95% CI (− 0.37, 0.04), p = 0.121] or parent-rated conduct problems (p > 0.05).<br><br>The cost per family of providing NFPP in the trial was significantly lower than IY (£1591 versus £2103). Although, there were no differences between NFPP and IY with regards clinical effectiveness, individually delivered NFPP cost less. However, this difference may be reduced when implemented in routine clinical practice.	
Lange et al., 2018 [17]	Children 3-7 with ADHD	RCT	BPT: New Forest Parenting Programme (NFPP)	TAU	164 (120/44)	Child ADHD symptoms (parent rating)	Child:<br>-Teacher ADHD RS-IV ratings.	ADHD Rating Scale-IV (ADHD-RS-IV)–Preschool Danish Version;	The parent training program was superior to TAU on parent-rated ADHD symptoms (p = .009; effect size d = 0.30) and on parenting self-efficacy and family strain.<br>Effects persisted to 36 weeks after treatment.	

							-Directly observed ADHD behaviors during solo play<br>-Conduct problems<br><br>Parent:<br>- Parental efficacy and satisfaction<br>- Parental stress<br>- Parental symptoms of ADHD<br>- Parent-child interactions	Child Solo Play instrument ;<br>Conduct scale of the Strengths and Difficulties Questionnaire (SDQ) ;<br>The Parenting Sense of Competence Scale;<br>Family Strain Index;<br>Adult ADHD Self-Report Scale (version 1.1) ;<br>Direct observation schedule; Global Impressions of Parent-Child Interactions;<br>General Health Questionnaire.	There were no effects on teacher ratings or direct observations of ADHD or on ratings of conduct problems or parenting.	
Abikoff et al., 2015 [18]	Preschool children 3.0-4.11 years with ADHD	RCT	Home-based BPT : New Forest Parenting Package (NFPP)	Home-based RCC	164 (121/43)	Child ADHD symptoms (teacher and parent ratings)	Clinician assessments of ADHD outcomes ; Laboratory measures of on-task behavior and delay of gratification.	Conners scales (parent, teacher); ADHD rating Scale- IV ; Children's levels of sustained and focused attention and activity during a videotaped five-minute period while playing with a standard multi-domain toy ('Play Park') coded by observers using a validated observational coding system; preschool version of the New York Teacher and Parent Rating Scales (NYTPRS) ; Delay of Gratification -Cookies Delay Task; Parenting Pratice Interview (PPI); Global Impressions of Parent Child Interactions - Revised (GIPCI-R) ; Parenting Stress Index - Short Form Revised (PSI-S); Consumer Satisfaction Questionnaire (CSQ).	NFPP was not superior to, and in some cases less effective than, HNC. Both treatments improved non-blind parent ratings of ADHD but not objective teacher and laboratory measures of ADHD.<br>Maintenance effects were obtained for some non-blind parent-reported outcomes, particularly for HNC.	
DuPaul et al., 2018 [19]	Preschool children with ADHD 3 years 0 month - 5 years 11 months	RCT	BPT: Face to Face (F2F)	Online BPT	47 (30/17)	Session completion and acceptability ;<br>Parent knowledge and parenting stress ;<br>Parent treatent fidelity;	N/A	Parent Stress Index - Short Form (PSI-SF); modified Intervention Rating Profile-15 (IRP-15); Conners Early Childhood Rating Scale	Both intervention formats resulted in high attendance (M = 80%) and significantly<br>improved parent knowledge of interventions, treatment implementation fidelity, and child behavior (reduced restlessness and impulsivity, improved self-control, affect, and mood) compared with WLC.<br><br>Parents in the F2F group reported significantly higher	

						Child behavior ratings		(CERS); Barkley Semistructured Diagnostic Interview ; Developmental Ability Scale-II (DAS-II); Social Communication Questionnaire (SCQ).	acceptability ratings.	
Hosainzadeh Maleki et al., 2014 [20]	Children 6-12 years with ADHD	RCT	BPT: Barkley's parent training group	Children Working memory training ;<br>BPT + Children Working memory training (combined group)	36 (“favors boys”)	Child ADHD symptoms	N/A	SNAP–IV<br>Child Behavior Checklist (CBCL)<br>Wechsler Intelligence Scale Fourth Edition (WISC-IV)	The three groups displayed significant differences in the following variables: attention deficit symptoms (F2)8&2)= 17.56, P<0.001, 2η=0.56), hyperactivity/impulsivity symptoms (F2)8 & 2)= 10.48, P<0.001, 2 η =0.43), total symptoms (F2) 8 &2)= 13.78, P<0.001, 2 η =0.50), attention problems (experience-based scales) (F2)8 & 2)= 10.39, P<0.001, 2 η =0.42), and ADHD symptoms based on DSM (F2)8 & 2)= 5.32, P<0.05, 2 η =0.28).<br><br>There was a significant difference (p< 0.05) in the decline of attention deficit and hyperactivity /impulsivity symptoms between the combined treatment group and working memory training group and also between the combined treatment group and the BPT group in SNAP.<br><br>In terms of attention problems (experience-based subscales) of CBCL, there was a significant difference (p< 0.001) between the combined treatment group and working memory training group.<br>Compared to the working memory training and parent training groups, the combined group demonstrated a significant decline (p< 0.01) in clinical symptoms of ADHD.<br><br>It was revealed that combined treatment in comparison with the other two methods suppressed the clinical symptoms of ADHD more significantly.	Very low
Yusuf et al., 2018 [21]	Children 7-12 years with ADHD receiving methylphenidate medication for at least 2 months	RCT	BPT: Triple P program	Waitlist	48 (38/10)	Child ADHD symptoms ;<br>Child ADHD severity ;<br>Child behavioral and emotional problems.	Parental attitudes ;<br>Family functioning.	Kiddie Schedule for Affective Disorders and Schizophrenia for School-Age Children – Present and Lifetime(K-SADS-PL); Strengths and Difficulties Questionnaire (SDQ); Children's Global Assessment Scale (CGAS); Clinical Global Impression- Severity Scale (CGI-S); DuPaul ADHD-RS-IV Inventory (DuPaul ADHD Scale); Parental Attitude Research Intrument (PARI); Family Assessment Device (FAD).	Differences of scale scores in the intervention group before and after Triple P:<br>Statistically significant decreases in DuPaul AD, DuPaul HA subscores, DuPaul total score and CGI-S and increase scores of CGAS after Triple P in intervention group (p < .001, p < .001, p < .001, p < .001, p < .001, respectively).<br><br>Statistically significant decrease in PARI overprotective parenting attitude (PARI OPA), PARI rejection of homemaking attitude (PARI RHRA), PARI strict discipline subscales.<br>Statistically significant increase in PARI democratic attitude subscale in the intervention group before and after Triple P (p = .001, p = .007, p = .008, p < .001, respectively).<br><br>Significant decreases in the scores of problem solving, communication, roles, affective emotions, affective attachment, behaviour control, general functionality subscales of FAD in the intervention group before and after Triple P (p = .020, p = .007, p < .001, p = .002, p < .001, p < .001, p < .001).<br><br>The waiting list group reported no significant changes after the waiting period.<br><br>Differences of scale scores between intervention and waiting list groups:<br>No statistical differences in subscales of PARI, except for democratic attitude subscale (Cohen’s d = 0.92) between the intervention group and the waiting list group after the Triple P.<br><br>No statistical differences between the intervention and waiting list groups in terms of all the subscales of FAD after Triple P (p > .05).	Very low

Behbahani et al., 2018 [23]	Children 7-12 years with ADHD	RCT	Mindful parenting training + pharmacotherapy (methylphenidate or risperidone)	Pharmacotherapy alone (methylphenidate or risperidone)	56 (37/19)	Child ADHD symptoms ; Parental stress.	N/A	Parenting Stress Index - Short form (PSI-SF); SNAP-IV.	There was a reduction in parenting stress (p < 0.001), negative parent-child interactions (p < 0.001), and children's problematic characteristics (p < 0.001) in the mindful parenting training group compared with the control group in the posttest and follow-up. Time and group effects were significant in all the subscales except in the subscales of the parents' disorders.<br><br>There was also a significant improvement in ADHD symptoms in the experimental group by comparison with the control group in the posttest (Attention deficit : p = 0.001 ; Hyperactivity : p = 0.04 ; Attention deficit and hyperactivity: p = 0.04) and follow-up (Attention deficit : p < 0.001; Hyperactivity : p < 0.001 ; Attention deficit and hyperactivity: p < 0.001).	Very low
									Statistically significant difference in SDQ emotional problems (Cohen's d = 0.99), SDQ ADHD problems (Cohen's d = 0.60) subscales and SDQ total scores (Cohen's d = 1.03), between intervention and waiting list group after Triple P (p = .004, p = .02, p = .001; respectively).	
Shafiee-Kandjani et al., 2017 [22]	Children 6-12 years with ADHD (mixed type)	RCT	Parent management training (PMT) + RCC	RCC	32 (32/0)	Child ADHD symptoms (parental ratings); Executive functions.		Kiddie Schedule for Affective Disorders and Schizophrenia for School-Age Children – Present and Lifetime (K-SADS-PL); Conner's Parents Rating Scale-Revised (CPRS); Continuous Performance Test (CPT); Go/ No Go Task; Raven's Progressive Matrices	There was a significant time-group interaction for hyperactivity (p = 0.032), opposition (p = 0.007), and total ADHD score (p = 0.010) of Conner's rating scale, and these scores had a decreasing pattern in PMT+ RCC group compared to children who received RCC alone.<br>This result was not achieved for inattention score.<br><br>Commission errors (reflecting impulsivity and response prevention), omission errors (reflecting inattention), and reaction time (reflecting vigilance) in Go/no Go test were not significantly different between the 2 groups.<br><br>Omission errors in CPT and the reaction time significantly improved in PMT+RCC group (P = 0.032).	Very low

Table S5. Detailed Information of School Based and Executive Studies.

Author/ Year	Population	Design	Intervention	Comparator	N (m/f)	Principal outcome	Other outcomes	Instruments	Findings	Grade
Corkum et al. 2015 [24]	Children Grade 1 to 6	RCT	Teacher help for ADHD group (web-based) (	Waitlist	58	ADHD symptoms	Impairment, Acceptability, Satisfaction	Conners 3rd Edition Parent and Teacher Rating Scales, Modi-	(Post treatment = 6 weeks, F-U = 6 weeks)<br><br>Conners teacher rated ADHD symptoms: Significant improvement in favor of intervention (λ = .84), F(2, 55) = 5.21, p = .008,	Low

			Schoolbased intervention)					fied version of Impariment rating scale (IRS, P +T)	η2 = .07, medium effect size). Clinical significance: More than one SD improvement for intervention group. It transposes in very elevated level of symptoms to elevated.<br>Conners parent rated ADHD symptoms: no significant group x time interaction. Main positive effect of time for all participants (p = 0.01), but not clinically significant (less than one SD).<br><br>Teacher impairment rating (IRS): Significant improvement (λ =<br>.86), F(2, 55) = 4.67, p = .01, η2 = .06, small to medium effect size) for intervention group only at post treatment and follow-up. No change in parent impairment rating.	
Evans et al. 2011 [25]	Grade 6-9 students (10-13 years middle school)	RCT	Challenging Horizons After-School Program (School oriented Executive function training and family-based intervention)	Community care Condition (contact information for local community resources.)	49	ADHD symptoms and impairment	Social and academic functioning, grades,	Disruptive Behavior Disorders Questionnaire (DBD), Impairment rating scale (IRS, T+P), Classroom Performance Survey (CPS), GPA	There is no significant condition x time interaction on Teacher rated DBD (Inattentive and hyperactivity symptoms) and on parent rating for inattentive symptoms. There is a positive condition x time interaction for parent rated DBD hyperactivity-impulsivity symptoms (t(163)= −3.37, p < .01). There is no difference between groups regarding GPA. No difference in impairment (IRS), except for teacher rated academic progress (time x condition interaction, t(213) =−2.04, p = .04). There is no report on clinical significance of results.	Very low
Pfiffner et al. 2014 [26]	7-11 years old, inattentive type	RCT	Child life and attention skills (CLAS) (Parent training, teacher consultation, child skills training)	Parent focused treatment (PFT),Treatment as usual (list of cimmunity treatment providers	199	DSM-IV Inattention symptoms	Organisation, Social skills, Functional impairment, Global psychosocial functioning	Child Symptom Inventory (CSI, inattention items, teacher and parent rated), Children's Organizational skills scale (COSS, T+P), Social Skills improvement sclae (SSIS, T+P), Impairment rating scale (T+P), 7-point clinical global impression scale	Post-treatment: All study arms improved in inattentive symptoms measurement (CSI-P, P=0.001; CSI-T, P=0.000). All post-hoc comparisons between CLAS and TAU group were significant. *Medium to very large effect size*: CSI-P (es=0.64), CSI-T(es=0.7), COSS-P (es=0.74), CGI-P(es=1.07), CGI-T (es=0.82). *Small to medium effect size*: COSS-T (es=0.49). *Small effect size* : SSIS-P (es=0.36), SSIS-T (es=0.34)). Post-hoc between CLAS and PFT. Near-medium or medium effect size: CSI-T, COSS-T, CGI-T. *Small effect size*: COSS-P, SSIS-T. *Non significant*: CSI-P, SSIS-P, CGI-P). Differences between active interventions were ns to medium and visible primarily at school. Follow-up (6 months): Parent report significant groups difference (CSI-P, p=0.000; COSS-P, p=0.007; CGI-P, p=0.001). Teacher did not report significant difference between groups (CSI-T, COSS-T, SSIS-T, CGI-T). Significant difference between CLAS and TAU. *Near-medium or medium effect size*: CSI-P, COSS-P, CGI-P. Significant difference between CLAS and PFT. *Small effect size*: COSS-P.<br><br>Clinically meaningful response rates (within one SD of norm for CSI; % of responders). CLAS (Post-treatment/Follow-up): Parent (54.8%/63%); Teacher (57.5%/47.2%).<br>PFT (P-T/F-U): P (43.2%/52.7%) ; T (44.4%/45.9%).<br>TAU (P-T/F-U): P (29.8%/36.2%) ; T (32.7%/38.8%). Summary: For parent-rated responders: significant difference between CLAS and TAU at post treatment and follow-up. For teacher-rated responders: significant difference at post treatment between CLAS and TAU, but not at follow-up.	low
Bikic et al. 2017 [27]	Children and adolescents from elementary to high school	MA	Organizational skills intervention (OST) (Various)	Parent education, waitlist or Treatment-as-usual. (various)	12 studies, 1054 children	Parent and Teacher-rated organizational skills	Inattention symptoms, academic performance.	Parent and Teacher-rated organizational skills (COSS, HPC - Homework problem checklist, HPQ - Homework performance questionnaire) and inattention symptoms (DBD - Diruptive behavior Disorders inattention subscale, CSI inattention subscale, Vanderbilt ADHD Diagnostic Parent Rating Scale inattention subscale, HPC inattention subscale, Pelham	Parent-reported inattention (n=893) : g = 0.56 (95% CI 0.38 to 0.74), medium effect) ; Teacher-reported inattention (n=590) : g = 0.26 (95% CI 0.01 to 0.52), small effect ; Parent-reported organizational skills (n=697): g= 0.83 (95% CI 0.32 to 1.34), large effect) ; Teacher-reported organizational skills (n=445): g= 0.54 (95% CI 0.17 to 0.91), medium effect ; Teacher-rated academic performance (n=663): g= 0.33 (95% CI 0.14 to 0.51), small effect. Student GPA (n=332) : g= 0.29 (95% CI 0.07 to 0.51), small effect.	Medium

								Questionnaire-IV in-attention subscale - SNAP-IV), Grade point average (GPA), Academic performance rating scale (APRS), Class-romm Performance Survey (CPS)		

References

1. Corbisiero, S.; Bitto, H.; Newark, P.; Abt-Mörstedt, B.; Elsässer, M.; Buchli-Kammermann, J.; Künne, S.; Nyberg, E.; Hofecker-Fallahpour, M.; Stieglitz, R.D. A Comparison of Cognitive-Behavioral Therapy and Pharmacotherapy vs. Pharmacotherapy Alone in Adults With Attention-Deficit/Hyperactivity Disorder (ADHD)-A Randomized Controlled Trial. *Front. Psychiatry* **2018**, *9*, 571. https://doi.org/10.3389/fpsyt.2018.00571.
2. Dittner, A.J.; Hodsoll, J.; Rimes, K.A.; Russell, A.J.; Chalder, T. Cognitive-behavioural therapy for adult attention-deficit hyperactivity disorder: a proof of concept randomised controlled trial. *Acta Psychiatr. Scand.* 2017, *137*, 125–137, https://doi.org/10.1111/acps.12836.
3. Safren, S.A.; Sprich, S.; Mimiaga, M.J.; Surman, C.; Knouse, L.; Groves, M.; Otto, M. Cognitive behavioral therapy vs relaxation with educational support for medication-treated adults with ADHD and persistent symptoms. *JAMA* **2010**, *304*, 875–880, https://doi.org/10.1001/jama.2010.1192.
4. Gu, Y.; Xu, G.; Zhu, Y. A Randomized controlled trial of mindfulness-based cognitive therapy for college students with ADHD. *J. Atten. Disord.* **2016**, *22*, 388–399, https://doi.org/10.1177/1087054716686183.
5. Emilsson, B.; Gudjonsson, G.; Sigurdsson, J.F.; Baldursson, G.; Einarsson, E.; Olafsdottir, H.; Young, S. Cognitive behaviour therapy in medication-treated adults with ADHD and persistent Symptoms: A randomized controlled trial. *BMC Psychiatry* **2011**, *11*, 116–116, https://doi.org/10.1186/1471-244x-11-116.
6. Schoenberg, P.L.; Hepark, S.; Kan, C.C.; Barendregt, H.P.; Buitelaar, J.K.; Speckens, A.E. Effects of mindfulness-based cognitive therapy on neurophysiological correlates of performance monitoring in adult attention-deficit/hyperactivity disorder. *Clin. Neurophysiol.* **2014**, *125*, 1407–1416, https://doi.org/10.1016/j.clinph.2013.11.031.
7. Solanto, M.V.; Marks, D.J.; Wasserstein, J.; Mitchell, K.; Abikoff, H.; Alvir, J.M.J.; Kofman, M.D. Efficacy of meta-cognitive therapy for adult ADHD. *Am. J. Psychiatry* **2010**, *167*, 958–968, https://doi.org/10.1176/appi.ajp.2009.09081123.
8. Solanto, M.V.; Surman, C.B.; Alvir, J. The efficacy of cognitive-behavioral therapy for older adults with ADHD: a randomized controlled trial. *Atten. Defic. Hyperact. Disord.* **2018**, *10*, 223–235. https://doi.org/10.1007/s12402-018-0253-1.
9. Cerrillo-Urbina, A.J.; García-Hermoso, A.; Sánchez-López, M.; Pardo-Guijarro, M.J.; Santos Gómez, J.L.; Martínez-Vizcaíno, V. The effects of physical exercise in children with attention deficit hyperactivity disorder: A systematic review and meta-analysis of randomized control trials.*Child: Care Health Develop.* **2015**, *41*, 779–788.
10. Zang, Y. Impact of physical exercise on children with attention deficit hyperactivity disorders: Evidence through a meta-analysis. *Medicine* **2019**, *98*, e17980. https://doi.org/10.1097/MD.0000000000017980.
11. Kang, K.D.; Choi, J.W.; Kang, S.G.; Han, D.H. Sports Therapy for attention, cognitions and sociality. *Laryngo-Rhino-Otologie* **2011**, *32*, 953–959, https://doi.org/10.1055/s-0031-1283175.
12. Meßler, C.F.; Holmberg, H.-C.; Sperlich, B. Multimodal therapy involving high-intensity interval training improves the physical fitness, motor skills, social behavior, and quality of life of boys with ADHD: A randomized controlled study. *J. Atten. Disord.* **2016**, *22*, 806–812, https://doi.org/10.1177/1087054716636936.
13. Rimestad, M.L.; Lambek, R.; Zacher Christiansen, H.; Hougaard, E. Short- and Long-Term Effects of Parent Training for Preschool Children With or at Risk of ADHD: A Systematic Review and Meta-Analysis. *J. Atten. Disord.* **2019**, *23*, 423–434. https://doi.org/10.1177/1087054716648775.
14. Mulqueen, J.M.; Bartley, C.A.; Bloch, M.H. Meta-Analysis: Parental Interventions for Preschool ADHD. *J. Atten. Disord.* **2015**, *19*, 118–124.
15. Herbert, S.D.; Harvey, E.A.; Roberts, J.L.; Wichowski, K.; Lugo-Candelas, C.I. A Randomized Controlled Trial of a Parent Training and Emotion Socialization Program for Families of Hyperactive Preschool-Aged Children. *Behav. Ther.* **2013**, *44*, 302–316, https://doi.org/10.1016/j.beth.2012.10.004.
16. Sonuga-Barke, E.J.S.; Barton, J.; Daley, D.; Hutchings, J.; Maishman, T.; Raftery, J.; Stanton, L.; Laver-Bradbury, C.; Chorozoglou, M.; Coghill, D.; et al. A comparison of the clinical effectiveness and cost of specialised individually delivered parent training for preschool attention-deficit/hyperactivity disorder and a generic, group-based programme: a multi-centre, randomised controlled trial of the New Forest Parenting Programme versus Incredible Years. *Eur. Child Adolesc. Psychiatry* **2017**, *27*, 797–809, https://doi.org/10.1007/s00787-017-1054-3.

17. Lange, A.-M.; Daley, D.; Frydenberg, M.; Houmann, T.; Kristensen, L.J.; Rask, C.; Sonuga-Barke, E.; Søndergaard-Baden, S.; Udupi, A.; Thomsen, P.H. Parent Training for Preschool ADHD in Routine, Specialist Care: A Randomized Controlled Trial. *J. Am. Acad. Child Adolesc. Psychiatry* **2018**, *57*, 593–602, https://doi.org/10.1016/j.jaac.2018.04.014.
18. Abikoff, H.B.; Thompson, M.; Laver-Bradbury, C.; Long, N.; Forehand, R.; Brotman, L.M.; Klein, R.G.; Reiss, P.; Huo, L.; Sonuga-Barke, E. Parent training for preschool ADHD: a randomized controlled trial of specialized and generic programs. *J. Child Psychol. Psychiatry* **2014**, *56*, 618–631, https://doi.org/10.1111/jcpp.12346.
19. DuPaul, G.J. Promoting success across school years for children with Attention-Deficit/Hyperactivity Disorder: Collaborative school-home intervention. *J. Am. Acad. Child. Adolesc. Psychiatry.* **2018**, *57*, 231–232. https://doi.org/10.1016/j.jaac.2018.02.001.
20. Hosainzadeh Maleki, Z.; Mashhadi, A.; Soltanifar, A.; Moharreri, F.; Ghanaei Ghamanabad, A. Barkley's Parent Training Program, Working Memory Training and their Combination for Children with ADHD: Attention Deficit Hyperactivity Disorder. *Iran J Psychiatry* **2014**, *9*, 47–54.
21. Yusuf, Ö.; Gonka, Ö.; Aynur, A.P. The effects of the triple P-positive parenting programme on parenting, family functioning and symptoms of attention-deficit/hyperactivity disorder. A randomized controlled trial. *Psychiatry Clin. Psychopharmacol.* **2018**, *29*, 665–673, https://doi.org/10.1080/24750573.2018.1542189.
22. Shafiee-Kandjani, A.R.; Noorazar, G.; Shahrokhi, H.; Nazari, M.A.; Farhang, S. Effect of Parent Management Training on Attention, Response Prevention, Impulsivity and Vigilance of Boys with Attention Deficient/Hyperactive Disorder. *Iran. J. Psychiatry Behav. Sci.* **2017**, *11*, https://doi.org/10.5812/ijpbs.4834.
23. Behbahani, M.; Zargar, F.; Assarian, F.; Akbari, H. Effects of Mindful Parenting Training on Clinical Symptoms in Children with Attention Deficit Hyperactivity Disorder and Parenting Stress: Randomized Controlled Trial. **2018**, *43*, 596–604, https://doi.org/10.30476/IJMS.2018.40582.
24. Corkum, P.; Elik, N.; Blotnicky-Gallant, P.A.C.; McGonnell, M.; McGrath, P. Web-Based Intervention for Teachers of Elementary Students With ADHD: Randomized Controlled Trial. *J. Atten. Disord.* **2015**, *23*, 257–269, https://doi.org/10.1177/1087054715603198.
25. Evans, S.W.; Schultz, B.K.; DeMars, C.E.; Davis, H. Effectiveness of the Challenging Horizons After-School Program for Young Adolescents With ADHD. *Behav. Ther.* 2011, *42*, 462–474, https://doi.org/10.1016/j.beth.2010.11.008.
26. Pfiffner, L.J.; Hinshaw, S.P.; Owens, E.; Zalecki, C.; Kaiser, N.M.; Villodas, M.; McBurnett, K. A two-site randomized clinical trial of integrated psychosocial treatment for ADHD-inattentive type.. *J. Consult. Clin. Psychol.* **2014**, *82*, 1115–1127, https://doi.org/10.1037/a0036887.
27. Bikic, A.; Reichow, B.; McCauley, S.A.; Ibrahim, K.; Sukhodolsky, D.G. Meta-Analysis of Organizational Skills Interventions for Children and Adolescents with Attention-Deficit/Hyperactivity Disorder. *Clin. Psychol. Rev.* **2017**, *52*, 108–123.
